# Supplementary material for: Immune response after oral immunization of goats and foxes with an NDV vectored rabies vaccine candidate
Source: PLoS Negl Trop Dis. 2024 Feb 26;18(2):e0011639. doi: 10.1371/journal.pntd.0011639 (PMC10919857; doi:10.1371/journal.pntd.0011639)
Supplement: S1 Data — Double underlined sequences present the NDV F gene end sequence at the 5’ end or the NDV HN gene start sequence at the 3’ end, respectively. Italic sequences present the intergenic regions, separating the genes from another. Dotted underlined sequences present the 5’ and 3’ non-coding regions (including the gene start and end sequences), that are derived from the NDV HN gene. The single underlined sequence presents the orf of SAD L16 glycoprotein. (DOCX) [file pntd.0011639.s001.docx]

**S1 Data: Nucleotide sequence (cDNA) of the additional transcriptional unit encoding the RABV-G open reading frame (orf) of rNDV_G_RABV_.** Double underlined sequences present the NDV F gene end sequence at the 5’ end or the NDV HN gene start sequence at the 3’ end, respectively. Italic sequences present the intergenic regions, separating the genes from another. Dotted underlined sequences present the 5’ and 3’ non-coding regions (including the gene start and end sequences), that are derived from the NDV HN gene. The single underlined sequence presents the orf of SAD L16 glycoprotein.

5‘-AGAAAAAA*CTACCGGTTGTAGATGACCAAAGGACGATAT*ACGGGTAGAACGGTAAGAGAGGCCGCCCCT

CAATTGCGAGCCAGGCTTCACAACCTCCGTTCTACCGCTTCACCGACAACAGTCCTCAACCATGGTTCCTCAGGCTCTCCTGTTTGTACCCCTTCTGGTTTTTCCATTGTGTTTTGGGAAATTCCCTATTTACACGATACCAGACAAGCTTGGTCCCTGGAGTCCGATTGACATACATCACCTCAGCTGCCCAAACAATTTGGTAGTGGAGGACGAAGGATGCACCAACCTGTCAGGGTTCTCCTACATGGAACTTAAAGTTGGATACATCTTAGCCATAAAAGTGAACGGGTTCACTTGCACAGGCGTTGTGACGGAGGCTGAAACCTACACTAACTTCGTTGGTTATGTCACAACCACGTTCAAAAGAAAGCATTTCCGCCCAACACCAGATGCATGTAGAGCCGCGTACAACTGGAAGATGGCCGGTGACCCCAGATATGAAGAGTCTCTACACAATCCGTACCCTGACTACCGCTGGCTTCGAACTGTAAAAACCACCAAGGAGTCTCTCGTTATCATATCTCCAAGTGTGGCAGATTTGGACCCATATGACAGATCCCTTCACTCGAGGGTCTTCCCTAGCGGGAAGTGCTCAGGAGTAGCGGTGTCTTCTACCTACTGCTCCACTAACCACGATTACACCATTTGGATGCCCGAGAATCCGAGACTAGGGATGTCTTGTGACATTTTTACCAATAGTAGAGGGAAGAGAGCATCCAAAGGGAGTGAGACTTGCGGCTTTGTAGATGAAAGAGGCCTATATAAGTCTTTAAAAGGAGCATGCAAACTCAAGTTATGTGGAGTTCTAGGACTTAGACTTATGGATGGAACATGGGTCTCGATGCAAACATCAAATGAAACCAAATGGTGCCCTCCCGATAAGTTGGTGAACCTGCACGACTTTCGCTCAGACGAAATTGAGCACCTTGTTGTAGAGGAGTTGGTCAGGAAGAGAGAGGAGTGTCTGGATGCACTAGAGTCCATCATGACAACCAAGTCAGTGAGTTTCAGACGTCTCAGTCATTTAAGAAAACTTGTCCCTGGGTTTGGAAAAGCATATACCATATTCAACAAGACCTTGATGGAAGCCGATGCTCACTACAAGTCAGTCAGAACTTGGAATGAGATCCTCCCTTCAAAAGGGTGTTTAAGAGTTGGGGGGAGGTGTCATCCTCATGTGAACGGGGTGTTTTTCAATGGTATAATATTAGGACCTGACGGCAATGTCTTAATCCCAGAGATGCAATCATCCCTCCTCCAGCAACATATGGAGTTGTTGGAATCCTCGGTTATCCCCCTTGTGCACCCCCTGGCAGACCCGTCTACCGTTTTCAAGGACGGTGACGAGGCTGAGGATTTTGTTGAAGTTCACCTTCCCGATGTGCACAATCAGGTCTCAGGAGTTGACTTGGGTCTCCCGAACTGGGGGAAGTATGTATTACTGAGTGCAGGGGCCCTGACTGCCTTGATGTTGATAATTTTCCTGATGACATGTTGTAGAAGAGTCAATCGATCAGAACCTACGCAACACAATCTCAGAGGGACAGGGAGGGAGGTGTCAGTCACTCCCCAAAGCGGGAAGATCATATCTTCATGGGAATCACACAAGAGTGGGGGTGAGACCAGACTGTAA*TTGAGTCAATTATACTTAAGGAGTTGGAAAGATGGCATTGTATCACCTATCTTCTGCGACATCAAGAATCAAACCGAATGCCGGCGCGTGCTCGAATTCCATGTTGCCAGTTGACCACAATCAGCCAGTGCTCATGCGATCAGATTAAGCCTTGTCAATAGTCTCTTGATTAAGAAAAAATGTAAGTGGCAATGCGATCGCTGACCAAAGGACGATAT*ACGGGTA-3‘
