# Supplementary material for: Immune response after oral immunization of goats and foxes with an NDV vectored rabies vaccine candidate
Source: PLoS Negl Trop Dis. 2024 Feb 26;18(2):e0011639. doi: 10.1371/journal.pntd.0011639 (PMC10919857; doi:10.1371/journal.pntd.0011639)
Supplement: S1 Fig — Quail muscle cells (QM9) were infected with rNDV or rNDV_GRABV (moi 0.01). Cell culture supernatants were harvested at indicated time points after infection (p. i.). Viral titers (TCID50/mL) were determined after titration on quail muscle (QM9) cells and subsequent immunostaining. Bar charts depict mean viral titers standard deviation (n = 4, two samples each from two independent experiments). (DOCX) [file pntd.0011639.s002.docx]

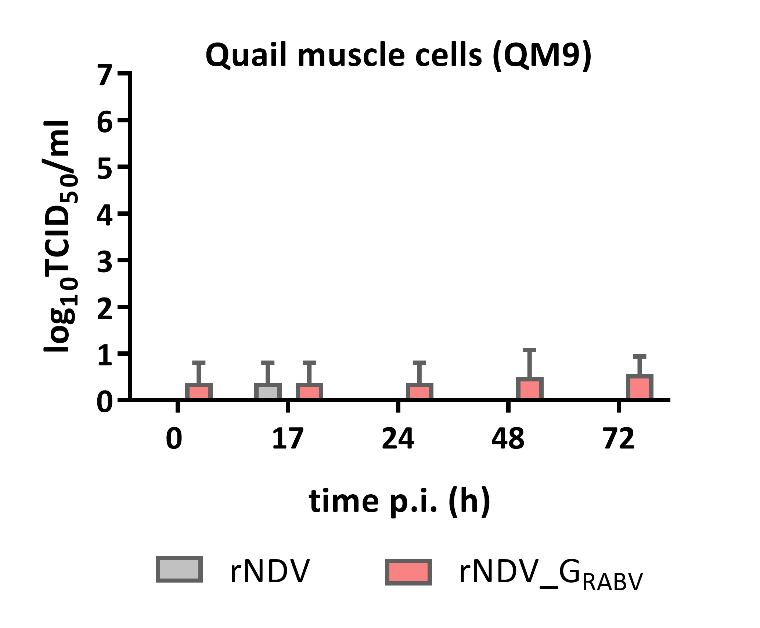


**S1 Fig. Addition to *in vitro* replication in cell lines originated from different species.** Quail muscle cells (QM9) were infected with rNDV or rNDV_G_RABV_ (moi 0.01). Cell culture supernatants were harvested at indicated time points after infection (p. i.). Viral titers (TCID_50_/mL) were determined after titration on quail muscle (QM9) cells and subsequent immunostaining. Bar charts depict mean viral titers standard deviation (n = 4, two samples each from two independent experiments).
