# Supplementary material for: Immune response after oral immunization of goats and foxes with an NDV vectored rabies vaccine candidate
Source: PLoS Negl Trop Dis. 2024 Feb 26;18(2):e0011639. doi: 10.1371/journal.pntd.0011639 (PMC10919857; doi:10.1371/journal.pntd.0011639)
Supplement: S2 Fig — Chicken embryo fibroblasts (DF-1) (A), bovine kidney cells (MDBK) and baby hamster kidney cells (BHK-21) (B) were infected with rNDV or rNDV_GRABV (multiplicity of infection (moi) 5) and harvested 24 h post infection (p. i.). Cell lysates and lysates of purified virions (10 μg per lane) (C) were subjected to SDS-PAGE and subsequently to Western blot analysis. Viral proteins were visualized by immunostaining with respective antibodies. Β-Actin was detected on every cell lysate blot as loading control. Blots of cell lysates are representative of three biological replicates. Blot of purified virions were done once. The figure depicts the uncompartmentalized illustrations of the western blots belonging to Fig 3 of the manuscript. (DOCX) [file pntd.0011639.s003.docx]

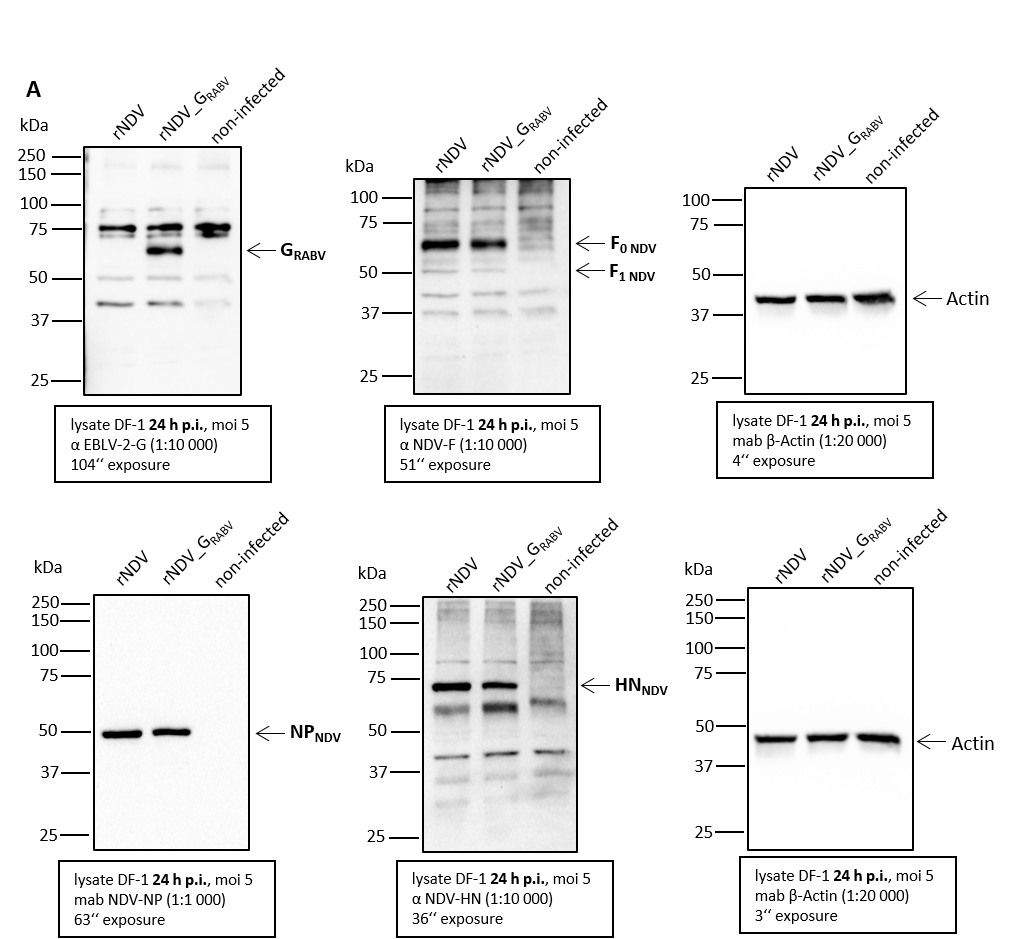


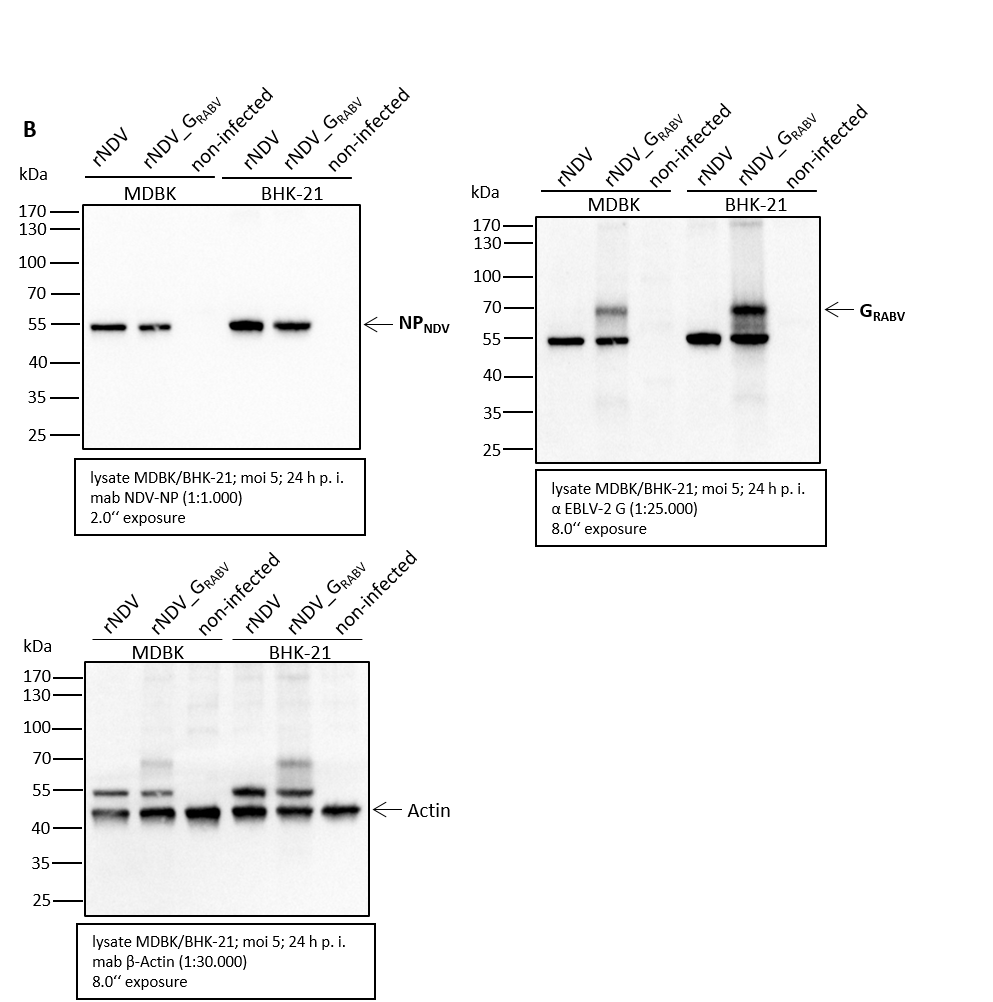


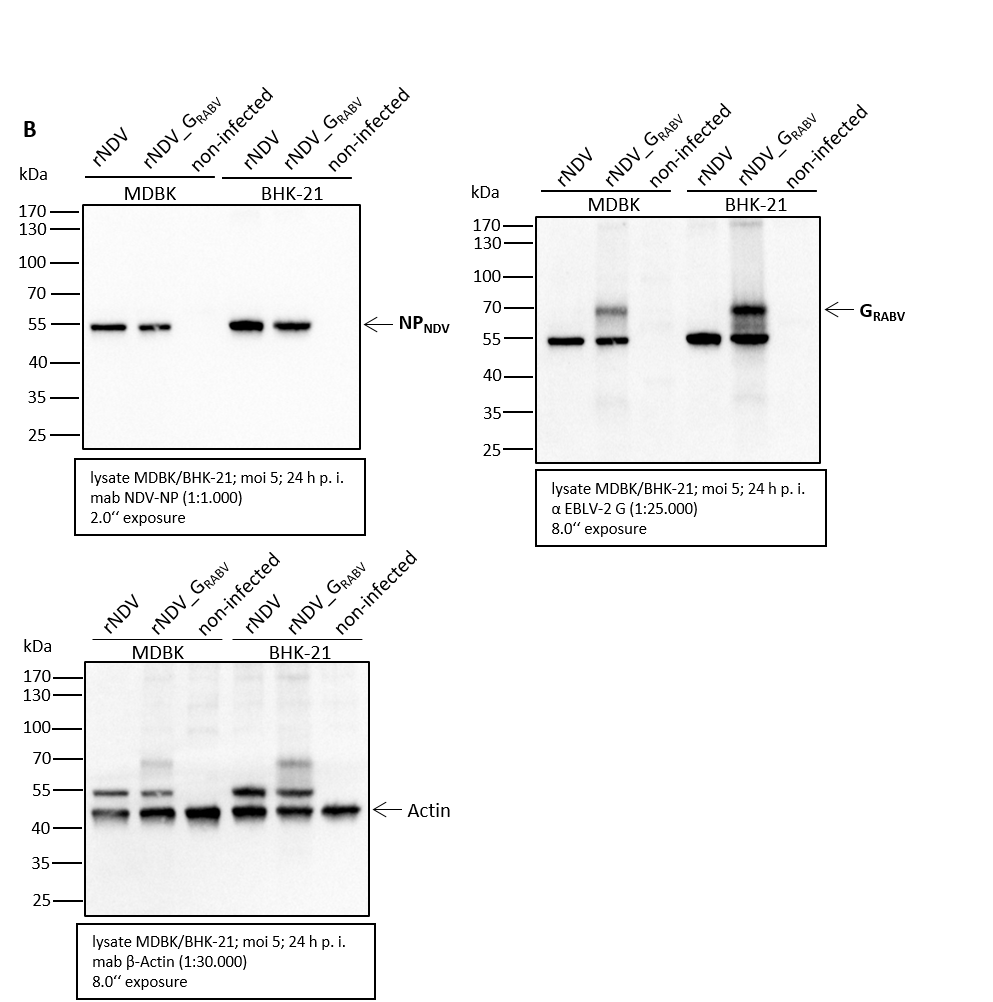


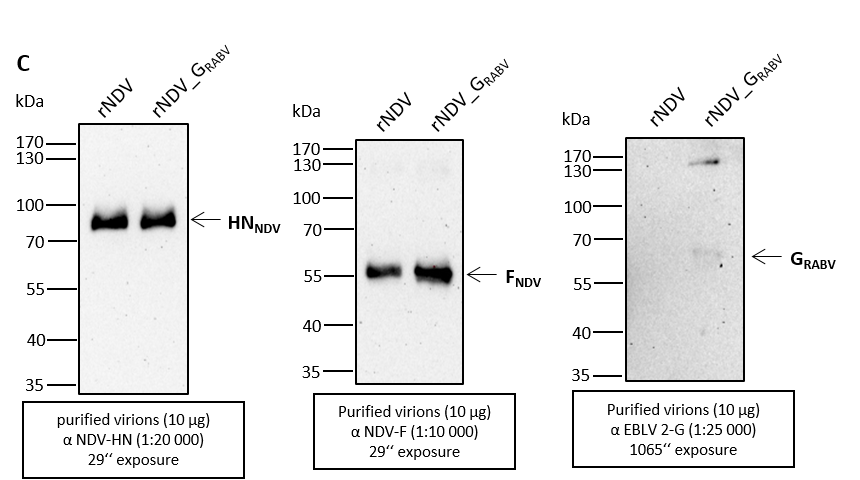


**S2 Fig**. **Addition to** *i****n vitro*-analysis of viral protein expression and virion composition.** Chicken embryo fibroblasts (DF-1) **(A)**, bovine kidney cells (MDBK) and baby hamster kidney cells (BHK-21) **(B)** were infected with rNDV or rNDV_G_RABV_ (multiplicity of infection (moi) 5) and harvested 24 h post infection (p. i.). Cell lysates and lysates of purified virions (10 µg per lane) **(C)** were subjected to SDS-PAGE and subsequently to Western blot analysis. Viral proteins were visualized by immunostaining with respective antibodies. Β-Actin was detected on every cell lysate blot as loading control. Blots of cell lysates are representative of three biological replicates. Blot of purified virions were done once. The figure depicts the uncompartmentalized illustrations of the western blots belonging to figure 3 of the manuscript.
