# Supplementary material for: Immune response after oral immunization of goats and foxes with an NDV vectored rabies vaccine candidate
Source: PLoS Negl Trop Dis. 2024 Feb 26;18(2):e0011639. doi: 10.1371/journal.pntd.0011639 (PMC10919857; doi:10.1371/journal.pntd.0011639)
Supplement: S3 Fig — (A) Goats and (B) foxes were directly orally vaccinated with either parental rNDV (n = 3) or RABV G expressing rNDV_GRABV (n = 6). Rectal temperature of goats and foxes as well as weight of foxes was monitored at indicated timepoints after oral vaccination. (DOCX) [file pntd.0011639.s004.docx]

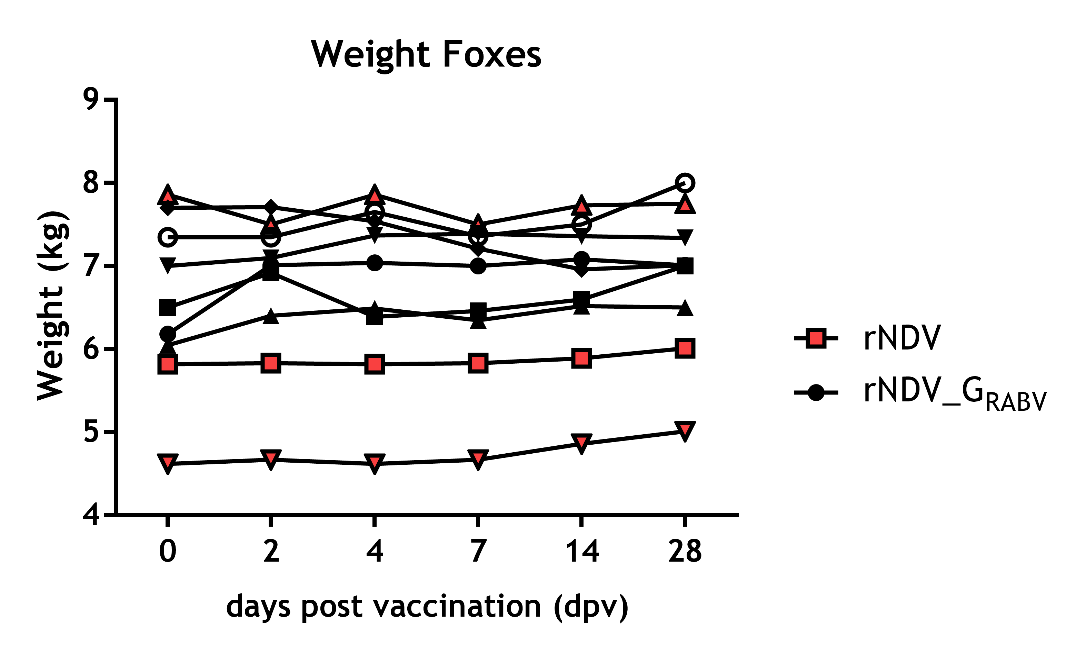

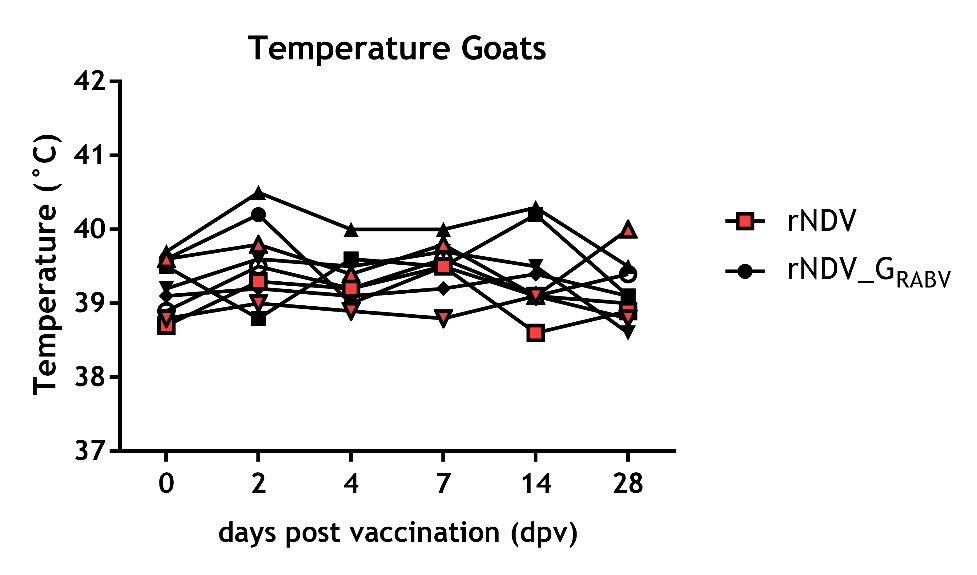


**A**


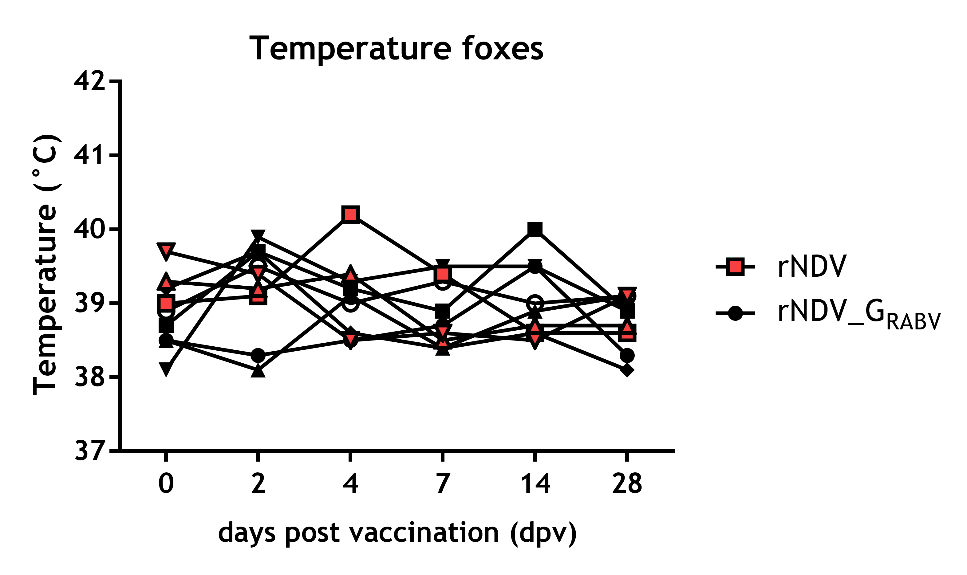

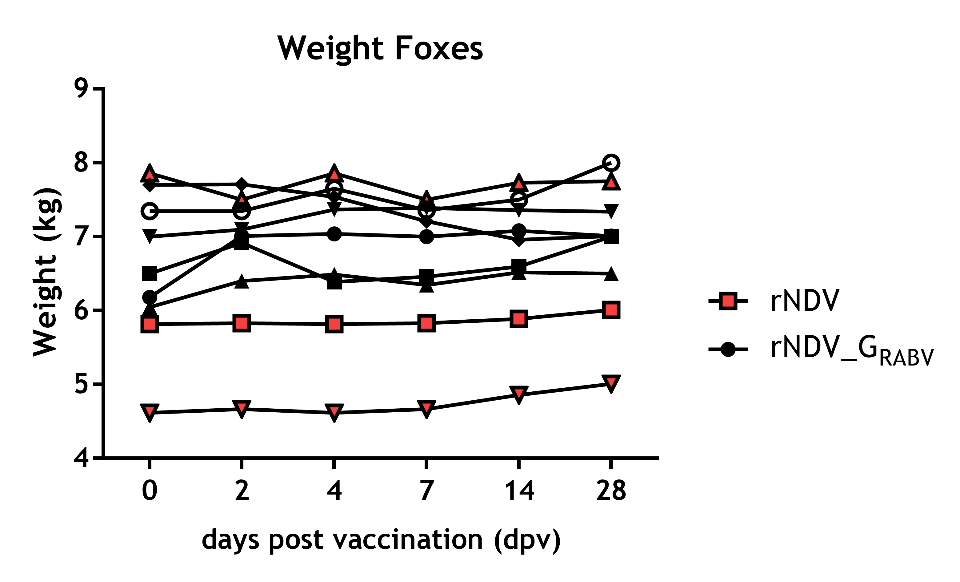


**B**

**S3 Fig.** **Development of temperature and weight after direct oral immunization. (A)** Goats and **(B)** foxes were directly orally vaccinated with either parental rNDV (n=3) or RABV G expressing rNDV_G_RABV_ (n=6). Rectal temperature of goats and foxes as well as weight of foxes was monitored at indicated timepoints after direct oral vaccination.
