# Supplementary material for: Immune response after oral immunization of goats and foxes with an NDV vectored rabies vaccine candidate
Source: PLoS Negl Trop Dis. 2024 Feb 26;18(2):e0011639. doi: 10.1371/journal.pntd.0011639 (PMC10919857; doi:10.1371/journal.pntd.0011639)
Supplement: S4 Fig — (A) Goats and (B) foxes were directly orally vaccinated with either parental rNDV (n = 3) or RABV G expressing rNDV_GRABV (n = 6). Sera of all animals was tested for NDV and RABV G specific antibodies at different timepoints after vaccination as described in material and methods section. Pearson correlation coefficient [-1; +1] was calculated to determine extent of correlation between serological assays in determining NDV or RABV specific antibodies. (DOCX) [file pntd.0011639.s005.docx]

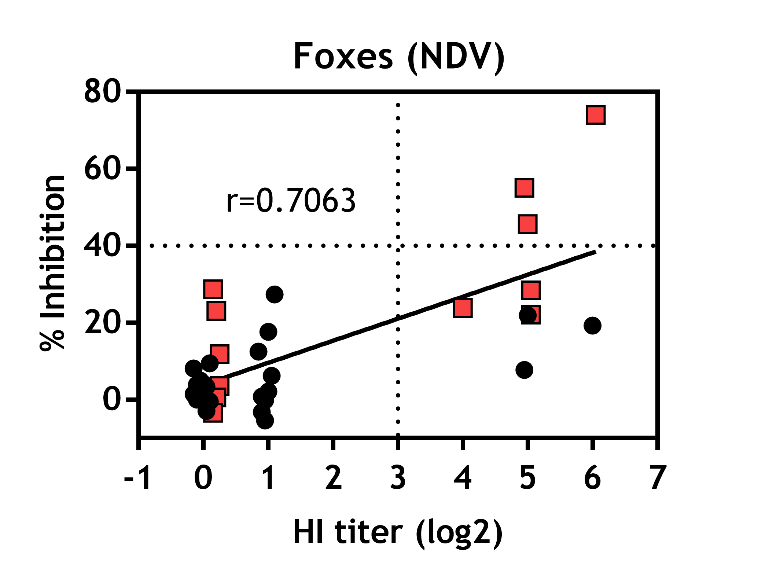

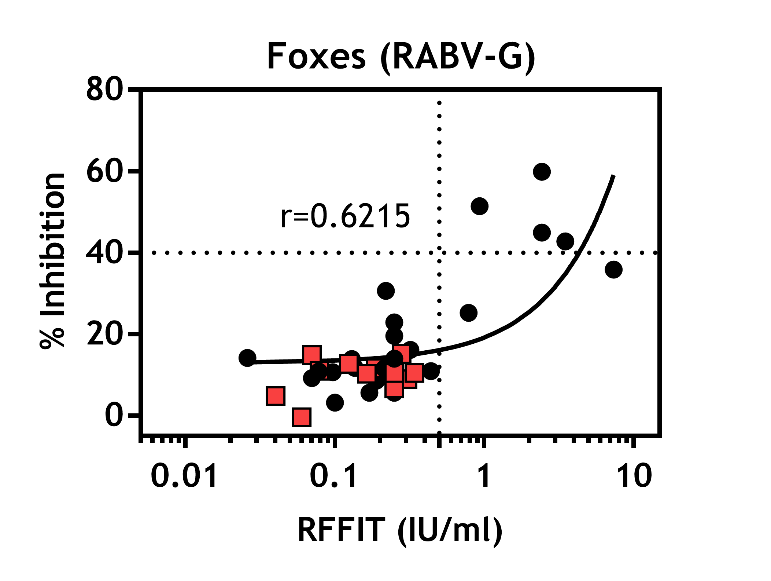

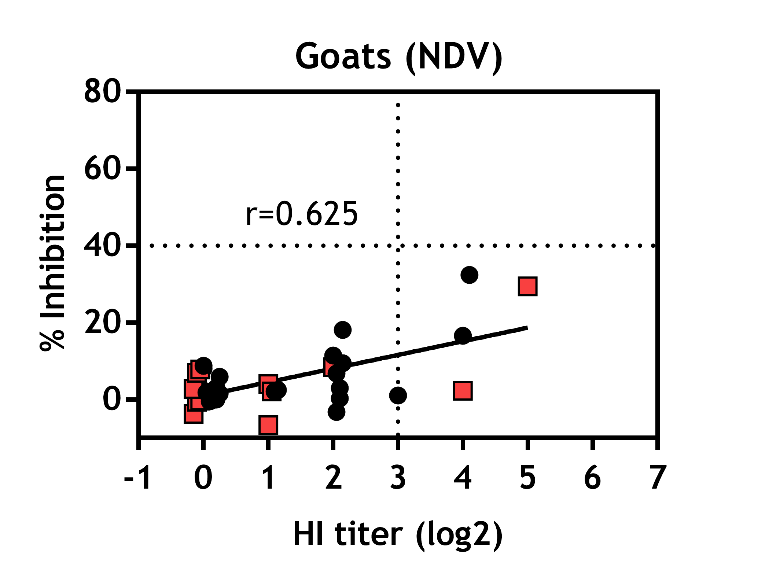

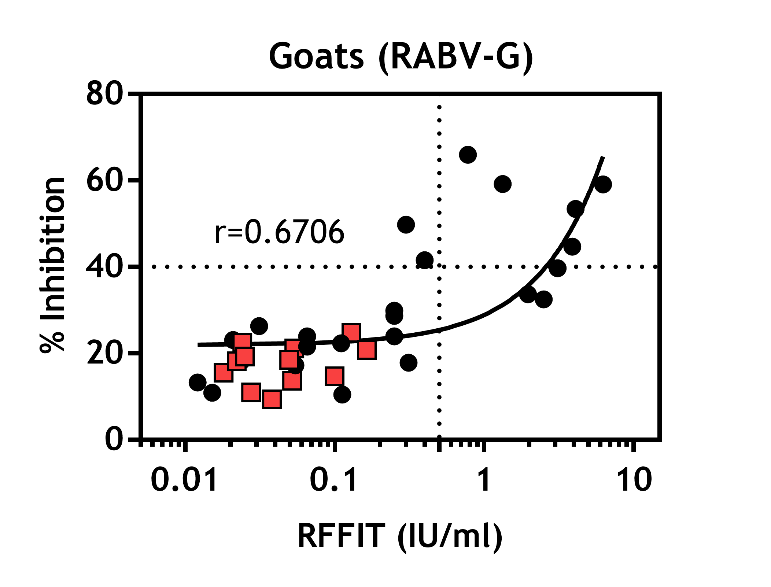

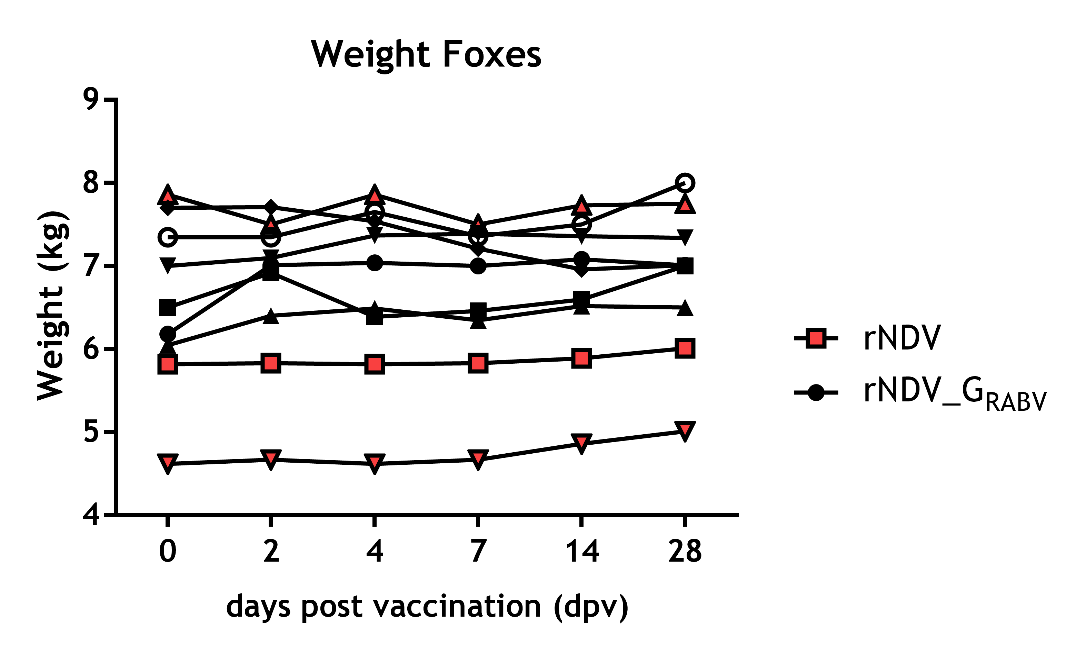


**S4 Fig.** **Correlation between NDV binding antibodies (ELISA) and hemagglutination inhibition antibodies (HI assay) or between RABV binding antibodies (ELISA) and RABV neutralizing antibodies (RFFIT) in oral vaccinated goats and foxes.** **(A)** Goats and **(B)** foxes were directly orally vaccinated with either parental rNDV (n=3) or RABV G expressing rNDV_G_RABV_ (n=6). Sera of all animals was tested for NDV and RABV G specific antibodies at different timepoints after vaccination as described in material and methods section. Pearson correlation coefficient [-1; +1] was calculated to determine extent of correlation between serological assays in determining NDV or RABV specific antibodies.
