# Supplementary material for: Immune response after oral immunization of goats and foxes with an NDV vectored rabies vaccine candidate
Source: PLoS Negl Trop Dis. 2024 Feb 26;18(2):e0011639. doi: 10.1371/journal.pntd.0011639 (PMC10919857; doi:10.1371/journal.pntd.0011639)
Supplement: S1 Table — Goats and foxes were directly orally vaccinated with either parental rNDV (n = 3) or RABV G expressing rNDV_GRABV (n = 6). Nasal, oral and rectal swabs were taken from all animals at indicated days after vaccination (dpv) and analyzed by quantitative real-time RT-PCR (RT-qPCR) for the presence of NDV NP specific RNA. Detected virus loads were indicated as genomic equivalents (GEQ) using calibration curves of defined RNA standards, which were included in each RT-qPCR run. (DOCX) [file pntd.0011639.s006.docx]

|  | | **0 dpv** | | | **2 dpv** | | | **4 dpv** | | | **7 dpv** | | | **14 dpv** | | |
| --- | --- | --- | --- | --- | --- | --- | --- | --- | --- | --- | --- | --- | --- | --- | --- | --- |
|  |  | nasal | oral | rectal | nasal | oral | rectal | nasal | oral | rectal | nasal | oral | recatl | nasal | oral | rectal |
| **rNDV** | G1 | N/A | N/A | N/A | 3,84E+02 | 8,47E+03 | N/A | N/A | N/A | N/A | N/A | N/A | N/A | N/A | N/A | N/A |
|  | G2 | N/A | N/A | N/A | N/A | N/A | N/A | N/A | N/A | N/A | N/A | N/A | N/A | N/A | N/A | N/A |
|  | G3 | N/A | N/A | N/A | N/A | N/A | 4,81E+03 | N/A | 3,94E+03 | N/A | N/A | N/A | N/A | N/A | N/A | N/A |
| **rNDV_G_RABV_** | G4 | N/A | N/A | N/A | N/A | 6,52E+03 | N/A | N/A | N/A | N/A | N/A | N/A | N/A | N/A | N/A | N/A |
|  | G5 | N/A | N/A | N/A | N/A | N/A | N/A | N/A | N/A | N/A | N/A | N/A | N/A | N/A | N/A | N/A |
|  | G6 | N/A | N/A | N/A | N/A | N/A | N/A | N/A | N/A | N/A | N/A | N/A | N/A | N/A | N/A | N/A |
|  | G7 | N/A | N/A | N/A | N/A | N/A | N/A | N/A | N/A | N/A | N/A | N/A | N/A | N/A | N/A | N/A |
|  | G8 | N/A | N/A | N/A | N/A | 6,07E+03 | N/A | N/A | N/A | N/A | N/A | N/A | N/A | N/A | N/A | N/A |
|  | G9 | N/A | N/A | N/A | N/A | N/A | N/A | N/A | N/A | N/A | N/A | N/A | N/A | N/A | N/A | N/A |
| **rNDV** | F9 | N/A | N/A | N/A | 3,38E+03 | 2,36E+04 | N/A | N/A | N/A | N/A | N/A | N/A | N/A | N/A | N/A | N/A |
|  | F8 | N/A | N/A | N/A | N/A | 3,30E+03 | N/A | N/A | N/A | N/A | N/A | N/A | N/A | N/A | N/A | N/A |
|  | F7 | N/A | N/A | N/A | 5,79E+03 | N/A | N/A | 5,46E+03 | 1,82E+05 | N/A | N/A | N/A | N/A | N/A | N/A | N/A |
| **rNDV_G_RABV_** | F6 | N/A | N/A | N/A | N/A | 2,76E+03 | N/A | N/A | 5,38E+03 | N/A | N/A | 2,52E+04 | N/A | N/A | N/A | N/A |
|  | F5 | N/A | N/A | N/A | 2,35E+03 | 4,83E+05 | N/A | N/A | N/A | N/A | N/A | N/A | N/A | N/A | N/A | N/A |
|  | F4 | N/A | N/A | N/A | N/A | 5,16E+04 | N/A | N/A | 3,45E+03 | N/A | N/A | 6,14E+04 | N/A | N/A | N/A | N/A |
|  | F3 | N/A | N/A | N/A | 2,21E+03 | N/A | N/A | N/A | N/A | N/A | N/A | N/A | N/A | N/A | N/A | N/A |
|  | F2 | N/A | N/A | N/A | N/A | 3,17E+04 | N/A | N/A | N/A | N/A | N/A | 3,14E+04 | N/A | N/A | N/A | N/A |
|  | F1 | N/A | N/A | N/A | N/A | N/A | N/A | N/A | 7,57E+05 | N/A | N/A | N/A | N/A | N/A | N/A | N/A |

**S1 Table.** **Individual virus replication and shedding after direct oral vaccination of goats and foxes.** Goats and foxes were directly orally vaccinated with either parental rNDV (n=3) or RABV G expressing rNDV_G_RABV_ (n=6). Nasal, oral and rectal swabs were taken from all animals at indicated days after vaccination (dpv) and analyzed by quantitative real-time RT-PCR (RT-qPCR) for the presence of NDV NP specific RNA. Detected virus loads were indicated as genomic equivalents (GEQ) using calibration curves of defined RNA standards, which were included in each RT-qPCR run.
