# Supplementary material for: Immune response after oral immunization of goats and foxes with an NDV vectored rabies vaccine candidate
Source: PLoS Negl Trop Dis. 2024 Feb 26;18(2):e0011639. doi: 10.1371/journal.pntd.0011639 (PMC10919857; doi:10.1371/journal.pntd.0011639)
Supplement: S2 Table — Goats and foxes were directly orally vaccinated with either parental rNDV (n = 3) or RABV G expressing rNDV_GRABV (n = 6). Serum was taken from all animals at indicated days after vaccination (dpv) and analyzed for antibodies specific to RABV by a competitive ELISA (cELISA; seropositivity: inhibition ≥ 40%) and the fluorescent focus inhibition test (RFFIT; seropositivity: IU/mL ≥ 0.5). Serum was analyzed for antibodies specific to NDV by a competitive ELISA (cELISA; seropositivity: inhibition ≥ 40%) and the hemagglutination inhibition (HI) assay (seropositivity: log2 ≥ 3). Seropositive samples are highlighted in green, indeterminate samples in orange. (DOCX) [file pntd.0011639.s007.docx]

**S2 Table.** **Individual serological data after direct oral vaccination of goats and foxes.** Goats and foxes were directly orally vaccinated with either parental rNDV (n=3) or RABV G expressing rNDV_G_RABV_ (n=6). Serum was taken from all animals at indicated days after vaccination (dpv) and analyzed for antibodies specific to RABV by a competitive ELISA (cELISA; seropositivity: inhibition ≥ 40 %) and the fluorescent focus inhibition test (RFFIT; seropositivity: IU/mL ≥ 0.5). Serum was analyzed for antibodies specific to NDV by a competitive ELISA (cELISA; seropositivity: inhibition ≥ 40 %) and the hemagglutination inhibition (HI) assay (seropositivity: log2 ≥ 3). Seropositive samples are highlighted in green, indeterminate samples in orange.

|  |  | **RABV** | | | | | | | | **NDV** | | | | | | | |
| --- | --- | --- | --- | --- | --- | --- | --- | --- | --- | --- | --- | --- | --- | --- | --- | --- | --- |
|  | | **0 dpv** | | **7 dpv** | | **14 dpv** | | **28 dpv** | | **0 dpv** | | **7 dpv** | | **14 dpv** | | **28 dpv** | |
|  |  | RFFIT  IU/ml | ELISA  %  Inhibition | RFFIT  IU/ml | ELISA  %  Inhibition | RFFIT  IU/ml | ELISA  %  Inhibition | RFFIT  IU/ml | ELISA  %  Inhibition | HI  log2 | ELISA  %  Inhibition | HI  log2 | ELISA  %  Inhibition | HI  log2 | ELISA  %  Inhibition | HI  log2 | ELISA  %  Inhibition |
| **rNDV** | G1 | 0,04 | 9,36 | 0,05 | 13,7 | 0,02 | 22,51 | 0,02 | 15,58 | 0 | -3,72 | 0 | 2,7 | 1 | 4 | 4 | 2,23 |
|  | G2 | 0,03 | 10,96 | 0,053 | 21,17 | 0,05 | 18,53 | 0,02 | 18,18 | 0 | -0,65 | 0 | 6,89 | 1 | 2,05 | 1 | -6,7 |
|  | G3 | 0,1 | 14,7 | 0,13 | 24,82 | 0,16 | 20,79 | 0,03 | 19,31 | 0 | -0,28 | 0 | 7,82 | 2 | 8,38 | 5 | 29,42 |
| **rNDV_G_RABV_** | G4 | 0,02 | 23,07 | 0,05 | 17,21 | 2,51 | 32,5 | 0,4 | 41,59 | 0 | 8,01 | 0 | 8,75 | 2 | 6,7 | 2 | 11,36 |
|  | G5 | 0,25 | 28,71 | 0,25 | 23,91 | 3,1 | 39,74 | 4,09 | 53,39 | 0 | 2,05 | 0 | 1,77 | 1 | 2,05 | 3 | 1,02 |
|  | G6 | 0,02 | 18,27 | 0,07 | 23,96 | 3,9 | 44,73 | 0,3 | 49,83 | 0 | 1,12 | 0 | -0,47 | 1 | 2,42 | 2 | -3,26 |
|  | G7 | 0,31 | 17,87 | 0,11 | 10,49 | 0,25 | 29,83 | 0,11 | 22,3 | 0 | -0,09 | 0 | 2,23 | 2 | 2,89 | 2 | 0,28 |
|  | G8 | 0,01 | 13,26 | 0,03 | 26,28 | 1,33 | 59,18 | 0,78 | 65,98 | 0 | 0 | 0 | 2,98 | 4 | 16,57 | 4 | 32,4 |
|  | G9 | 0,02 | 10,9 | 0,07 | 21,57 | 1,97 | 33,67 | 6,25 | 59,14 | 0 | 1,49 | 0 | 5,87 | 2 | 9,4 | 2 | 18,06 |
| **rNDV** | F9 | 0,31 | 8,92 | 0,07 | 14,84 | 0,08 | 10,95 | 0,12 | 12,66 | 0 | 3,45 | 0 | 11,82 | 5 | 22,07 | 5 | 28,4 |
|  | F8 | 0,06 | -0,49 | 0,19 | 11,62 | 0,25 | 10,48 | 0,16 | 10,23 | 0 | 0,56 | 0 | 23 | 5 | 45,62 | 4 | 23,84 |
|  | F7 | 0,04 | 4,75 | 0,25 | 6,7 | 0,28 | 15,09 | 0,34 | 10,44 | 0 | -3,45 | 0 | 28,68 | 5 | 55,03 | 6 | 74,02 |
| **rNDV_G_RABV_** | F6 | 0,19 | 8,53 | 0,25 | 19,56 | 7,38 | 35,86 | 2,44 | 59,87 | 0 | -0,47 | 0 | 9,4 | 1 | 27,28 | 5 | 21,88 |
|  | F5 | 0,10 | 3,19 | 0,22 | 11,57 | 0,79 | 25,17 | 0,22 | 30,63 | 0 | 3,26 | 0 | -2,98 | 1 | 6,15 | 1 | 2,05 |
|  | F4 | 0,44 | 10,92 | 0,32 | 16,1 | 3,51 | 42,76 | 0,93 | 51,4 | 0 | 4,1 | 0 | 0,84 | 1 | 17,6 | 6 | 19,18 |
|  | F3 | 0,03 | 14,16 | 0,1 | 10,61 | 0,13 | 13,89 | 0,08 | 10,76 | 0 | 4,93 | 0 | 4,84 | 1 | -0,19 | 1 | -5,49 |
|  | F2 | 0,17 | 5,61 | 0,14 | 11,73 | 0,25 | 13,98 | 0,07 | 9,14 | 0 | 0 | 0 | 3,91 | 1 | 0,74 | 1 | -3,26 |
|  | F1 | 0,25 | 5,56 | 0,25 | 13,48 | 0,25 | 22,86 | 2,43 | 44,92 | 0 | 1,4 | 0 | 8,1 | 1 | 12,48 | 5 | 7,64 |
